# Complete resection of a rectal post-endoscopic-resection residual tumor including four endoclips using underwater endoscopic mucosal resection

**DOI:** 10.1055/a-2155-6217

**Published:** 2023-09-28

**Authors:** Kosei Hashimoto, Yoshikazu Hayashi, Takaaki Morikawa, Masahiro Okada, Takahito Takezawa, Atsushi Kihara, Hironori Yamamoto

**Affiliations:** 1 Department of Medicine, Division of Gastroenterology, Jichi Medical University, Shimotsuke, Japan; 2 Department of Pathology, Jichi, Medical University, Shimotsuke, Japan

Endoscopic submucosal dissection (ESD) can facilitate complete removal of residual tumors even after a failed endoscopic resection and even when endoclips are left in place 1 . However, ESD requires advanced endoscopic skills, long procedure times, and expensive devices. Underwater endoscopic mucosal resection (UEMR) has recently emerged as a game-changing technique for endoscopic polyp resection. UEMR is usually simpler, cheaper, and more reliable than the conventional endoscopic resection techniques. Additionally, UEMR could even assist resection of stage T1b lesions 2 and residual/recurrent colorectal lesions 3 . We illustrate the use of the UEMR technique for the complete endoscopic resection of a residual rectal tumor, including four endoclips, after endoscopic mucosal resection (EMR).

A 72-year-old woman was referred for a suspected residual tumor after conventional EMR in the distal rectum; intramucosal cancer with a positive horizontal margin was identified on histopathological assessment. Outpatient colonoscopy revealed a 10-mm residual lesion with four endoclips remaining from the previous EMR. Magnifying narrow-band light examination suggested a low-grade adenoma ( Fig. 1 , Video 1 ). Endoscopic ultrasonography did not clearly demonstrate the submucosa under the lesion because of the acoustic shadow of the endoclips. When snaring the entire lesion under water immersion was attempted, the endoclips surely moved up on the snared protruding mucosa ( Fig. 2 ). This suggests that complete endoscopic resection, including the endoclips, using UEMR for recovery is both safe and feasible on an outpatient basis. The UEMR was completed without any complications. Pathological evaluation revealed a well-differentiated adenocarcinoma with no lymphovascular invasion and negative margins ( Fig. 3 ). The muscularis mucosa was injured by the endoclips and was obscured in the pathological specimen.

**Fig. 1:**
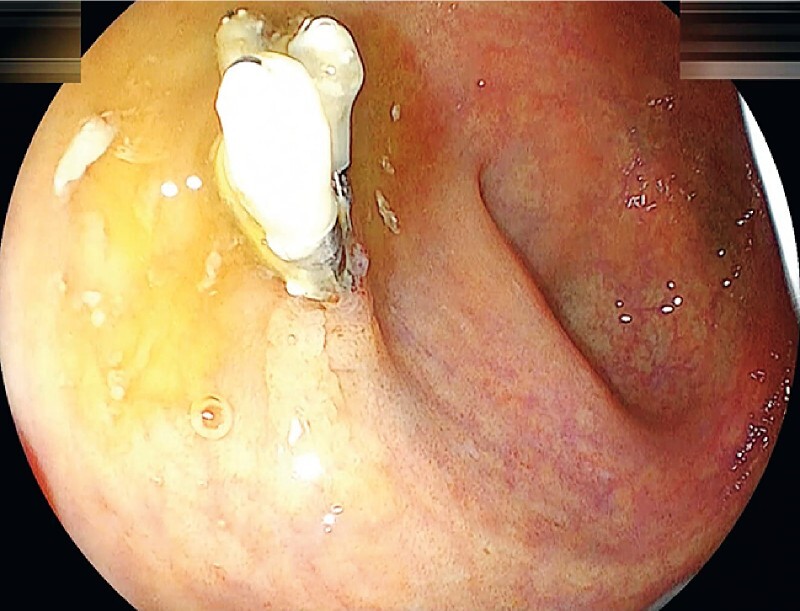
The lesion was examined using a magnifying narrow-band light colonoscopy (EC-760ZP-W/M; Fujifilm, Tokyo, Japan). A 10-mm scarred sessile tumor with four endoclips was observed in the distal rectum.

**Video 1** Complete resection of a rectal post-endoscopic-resection residual tumor including four endoclips using underwater endoscopic mucosal resection.


**Fig. 2:**
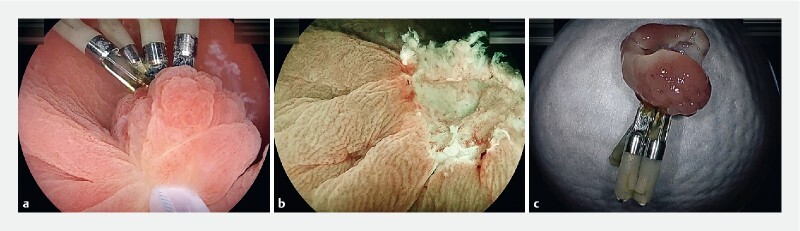
Sequential pictures of the underwater endoscopic mucosal resection (UEMR) of the tumor. The tip of the snare (15-mm Rota snare; Medi-Globe GmbH, Achenmühle, Germany) was securely placed at the normal mucosa beyond the tumor with a sufficient proximal margin. The snare was gradually closed twice until it captured the entire tumor with its surrounding normal mucosa while aspirating the water. **a** After the snare was closed, the tumor with endoclips was completely within the area captured inside the snare. Four endoclips were away from the snared point. The secured lesion was cut using pure-cut mode diathermy (ESG-100; Olympus, Tokyo, Japan). **b** No residual lesion was identified around the mucosal defect. An endoscopic en bloc resection was performed. The mucosal defect was closed using two reopenable clips (Sureclip Plus, Micro-Tech Co. Ltd., Nanjing, China) and two endoclips (EZ-clip, Olympus). **c** Resected specimens. The UEMR was completed without any complications.

**Fig. 3:**
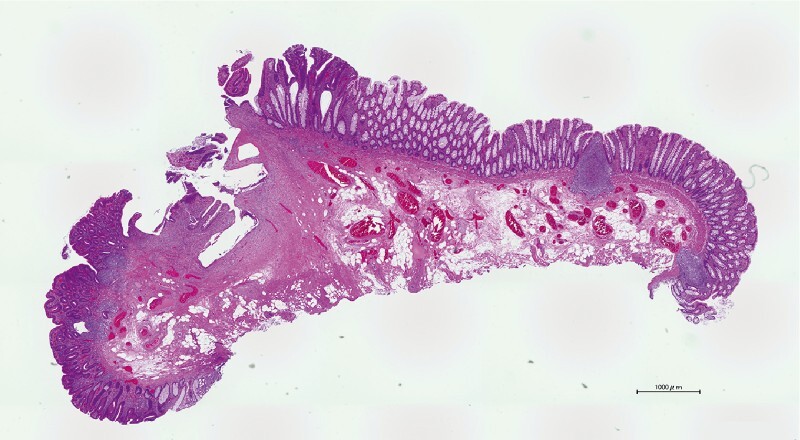
Pathology. Hematoxylin and eosin-stained specimen (40 × magnification). Well-differentiated adenocarcinoma was limited to the shallow submucosa with no lymphovascular invasion and negative margins.

This case demonstrates that a residual tumor with four endoclips still in place after EMR can be safely and completely resected using UEMR.

Endoscopy_UCTN_Code_TTT_1AQ_2AC

## Notes

Endoscopy E-Videos

https://eref.thieme.de/e-videos
*E-Videos* is an open access online section of the journal *Endoscopy* , reporting on interesting cases and new techniques in gastroenterological endoscopy. All papers include a high-quality video and are published with a Creative Commons CC-BY license. Endoscopy E-Videos qualify for HINARI discounts and waivers and eligibility is automatically checked during the submission process. We grant 100% waivers to articles whose corresponding authors are based in Group A countries and 50% waivers to those who are based in Group B countries as classified by Research4Life (see: https://www.research4life.org/access/eligibility/ ). This section has its own submission website at https://mc.manuscriptcentral.com/e-videos

## References

[1] Yamashita S Sunada K Yamamoto H Pocket-creation method enables colorectal endoscopic submucosal dissection for local recurrence with residual endoclips Dig Endosc 2021 33 e31 e33 10.1111/den.13900 33368643

[2] Fukuda H Takeuchi Y Shoji A Curative value of underwater endoscopic mucosal resection for submucosally invasive colorectal cancer J Gastroenterol Hepatol 2021 36 2471 2478 10.1111/jgh.15513 33788311

[3] Ohmori M Yamasaki Y Iwagami H Propensity score-matched analysis of endoscopic resection for recurrent colorectal neoplasms: A pilot study J Gastroenterol Hepatol 2021 36 2568 2574 10.1111/jgh.15519 33843099

